# Treino de Força Reduz Stress Oxidativo Cardíaco e Renal em Ratos com Hipertensão Renovascular

**DOI:** 10.36660/abc.20190391

**Published:** 2021-01-27

**Authors:** Rodrigo Miguel-dos-Santos, Jucilene Freitas dos Santos, Fabricio Nunes Macedo, Anderson Carlos Marçal, Valter J. Santana, Rogerio Brandão Wichi, Sandra Lauton-Santos

**Affiliations:** 1 Norwegian University of Science and Technology Cardiac Exercise Reserch Group Department of Circulation and Medical Imaging Trondheim Noruega Norwegian University of Science and Technology - Cardiac Exercise Reserch Group, Department of Circulation and Medical Imaging, Trondheim - Noruega; 2 Programa de Pós-Graduação em Educação Física Universidade Federal de Sergipe São CristóvãoSE Brasil Programa de Pós-Graduação em Educação Física, Universidade Federal de Sergipe, São Cristóvão, SE – Brasil; 3 Programa de Pós-Graduação em Ciências Fisiológicas Universidade Federal de Sergipe São CristóvãoSE Brasil Programa de Pós-Graduação em Ciências Fisiológicas, Universidade Federal de Sergipe, São Cristóvão, SE – Brasil; 4 Instituto de Ciências Biológicas e da Saúde Universidade Federal de Alagoas MaceióAL Brasil Instituto de Ciências Biológicas e da Saúde, Universidade Federal de Alagoas,Maceió, AL – Brasil; 5 Departamento de Educação Física Centro Universitário Estácio de Sergipe AracajuSE Brasil Departamento de Educação Física, Centro Universitário Estácio de Sergipe, Aracaju, SE – Brasil; 6 Departamento de Morfologia Universidade Federal de Sergipe São CristóvãoSE Brasil Departamento de Morfologia da Universidade Federal de Sergipe, São Cristóvão, SE – Brasil; 7 Programa de Pós-Graduação em Medicina Universidade Federal de Sergipe São CristóvãoSE Brasil Programa de Pós-Graduação em Medicina, Universidade Federal de Sergipe, São Cristóvão, SE – Brasil

**Keywords:** Hipertensão Renovascular, Treinamento de Resistência, Antioxidantes, Estresse Oxidativo, Obstrução da Artéria Real, Oxidação-Redução

## Abstract

**Fundamento:**

O treino de força tem efeitos benéficos em doenças renais, além de ajudar a melhorar a defesa antioxidante em animais saudáveis.

**Objetivo:**

Verificar se o treino de força reduz o dano oxidativo ao coração e rim contralateral para cirurgia de indução de hipertensão renovascular, bem como avaliar as alterações na atividade das enzimas antioxidantes endógenas superóxido dismutase (SOD), catalase (CAT) e glutationa peroxidase (GPx).

**Métodos:**

Dezoito ratos machos foram divididos em três grupos (n=6/grupo): placebo, hipertenso e hipertenso treinado. Os animais foram induzidos a hipertensão renovascular através da ligação da artéria renal esquerda. O treino de força foi iniciado quatro semanas após a indução da hipertensão renovascular, teve 12 semanas de duração e foi realizada a 70% de 1RM. Depois do período de treino, os animais foram submetidos a eutanásia e o rim esquerdo e o coração foram retirados para realizar a quantificação de peróxidos de hidrogênio, malondialdeído e grupos sulfidrílicos, que são marcadores de danos oxidativos. Além disso, foram medidas as atividades das enzimas antioxidantes superóxido dismutase, catalase e glutationa peroxidase. O nível de significância adotado foi de 5% (p < 0,05).

**Resultados:**

Depois do treino de força, houve redução de danos oxidativos a lipídios e proteínas, como pode-se observar pela redução de peróxidos de hidrogênio e níveis sulfidrílicos totais, respectivamente. Além disso, houve um aumento nas atividades das enzimas antioxidantes superóxido dismutase, catalase e glutationa peroxidase.

**Conclusão:**

O treino de força tem o potencial de reduzir danos oxidativos, aumentando a atividades de enzimas antioxidantes. (Arq Bras Cardiol. 2021; 116(1):4-11)

## Introdução

A hipertensão renovascular, um tipo de hipertensão causada por estenose arterial renal total ou parcial devido a fatores genéticos ou aterosclerose, é uma causa importante de hipertensão secundária.^1^ Nesse tipo de hipertensão, o aumento da pressão arterial (PA) é iniciado por uma maior liberação de renina pelo rim isquêmico resultante do fluxo sanguíneo para esse órgão, devido à estenose da artéria renal.^1 , 2^

A renina é responsável pela conversão de angiotensinogênio em angiotensina I, que é clivada pela enzima conversora da angiotensina (ECA) produzindo angiotensina II (Ang II).^3 , 4^ Portanto, a elevação da renina acarreta um aumento na liberação de Ang II. A Ang II, por sua vez, ativa as enzimas NADPH oxidase^3^ e xantina oxidase,^4^ aumentando a produção de ânion superóxido (O_2_^-^), uma molécula sinalizadora de pró-oxidante altamente reativa que pode causar danos oxidativos a lipídios, proteínas e DNA, e foi descrita na hipertensão renovascular.^5 , 6^ O aumento dos danos oxidativos no rim e no coração pode levar ao amento de fibrose do tecido, causando a redução de sua função,^2^ e, acabando por resultar em falência do rim que não foi afetado por estenose e disfunção cardíaca.

Está relatada, na literatura, a ação protetora do treino de força no tratamento de várias doenças, entre as quais está a hipertensão arterial.^7 , 8^ Entre os benefícios gerados pelo treino de força, já se observou que ela promove a melhoria da função cardíaca,^9^ bem como o aumento da atividade e/ou expressão das enzimas envolvidas na síntese de óxido nítrico.^10 , 11^ Essas mudanças causam o aumento da liberação de óxido nítrico, melhoria do tônus vascular,^10 , 11^ e redução da PA em animais normotensos^12^ e hipertensos.^13^

Além disso, também foi descrita a ação protetora do treino de força no stress oxidativo, melhorando a defesa antioxidante no fígado^14^ e no músculo esquelético.^15^ Entretanto, os efeitos do treino de força no coração e no rim contralateral para estenose arterial renal não são conhecidos. Dessa forma, o objetivo deste estudo foi verificar se o treino de força reduz o dano oxidativo ao coração e rim contralateral para cirurgia de indução de hipertensão renovascular, bem como avaliar as alterações na atividade das enzimas antioxidantes endógenas superóxido dismutase (SOD), catalase (CAT) e glutationa peroxidase (GPx).

## Métodos

O protocolo experimental do presente estudo foi aprovado pelo Comitê de Ética em Pesquisa Animal (CEPA - Nº 54/2015) da Universidade Federal de Sergipe, em conformidade com os Princípios Éticos da Experimentação Animal adotados pelo Conselho Nacional de Controle de Experimentação Animal (CONCEA).

### Amostra

Ratos Wistar machos, com idade de 10 a 12 semanas e massa corporal entre 240 e 270 g, foram obtidos pela instalação de animais da Universidade Federal de Sergipe. Os animais foram alojados em gaiolas coletivas (cinco animais/gaiola), mantidos em condições controladas de temperatura (23 ± 1ºC) e um ciclo claro-escuro de 12 horas, com alimentação e água à vontade.

### Grupos experimentais

Dezoito animais foram divididos aleatoriamente, por meio de um software online, em três grupos experimentais (n = 6 por grupo): placebo, hipertensos e hipertensos treinados. O tamanho da amostra foi definido por conveniência.

### Indução da hipertensão renovascular

A indução da hipertensão foi realizada em animais dos grupos dos hipertensos e dos hipertensos treinados, pelo modelo de clipagem arterial desenvolvido por Goldblatt et al.,^16^ seguindo as adaptações propostas por Cangiano et al., ^17^ Assim, com os animais em anestesia profunda (cetamina 90 mg/kg e xilazina 10 mg/kg, intraperitoneal), foi feita uma incisão no flanco esquerdo da traseira do animal para exteriorização do rim esquerdo, e foi feita uma ligação de artéria renal com uma linha cirúrgica de algodão estéril 4,0. Os animais do grupo de placebo passaram por cirurgia apenas para exteriorização do rim esquerdo para reproduzir o stress gerado pela cirurgia nos animais dos grupos dos hipertensos e dos hipertensos treinados. Todos os animais receberam analgésicos (Flunixina meglumina, sc, 1 mg/Kg, a cada 24 horas) por quatro dias depois do pós-cirúrgico.

### Protocolo do treino de força

Três semanas após a cirurgia de indução da hipertensão, os animais dos grupos dos hipertensos e dos hipertensos treinados foram adaptados ao equipamento de treino por cinco dias, mantendo os animais presos aos equipamentos durante 10 minutos por dia. Daí em diante, um teste de repetição máxima (1RM) foi realizado nos animais de ambos os grupos e a cada duas semanas no grupo dos hipertensos treinados, para determinar a carga usada nas sessões de musculação. O teste foi realizado novamente no grupo hipertenso sedentário apenas ao final do protocolo experimental.

Os testes de repetição máxima foram realizados segundo as diretrizes do *American College of Sports Medicine* (Colégio Americano de Medicina Esportiva)^18^ para seres humanos, com três tentativas por teste. O primeiro teste 1RM foi realizado com 3x o peso corporal do animal, ajustando para cima ou para baixo para a tentativa seguinte, dependendo do desempenho do animal na tentativa. Os animais puderam descansar por três minutos entre cada tentativa.

**Figura 1:**
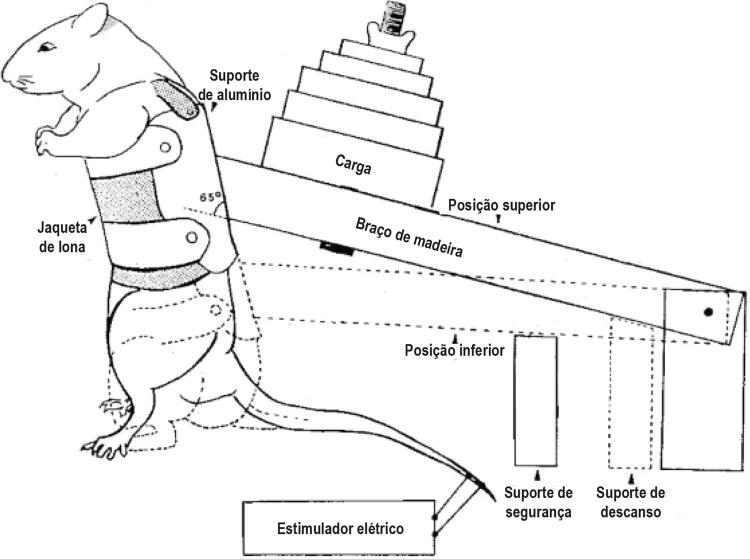
– Ilustração representativa do aparelho para treino de força. (Adaptado de Tamaki et al., 1992).

O treino de força foi realizado conforme descrito por Tamaki, Uchiyama e Nakano,^19^ e conforme já usado em outros estudo.^20 - 22^ Resumidamente, esse modelo de treino de força é realizado em um aparelho que simula o agachamento onde o torso do rato é equipado com uma jaqueta de lona, que o mantém na posição ereta ( Figura 1 ). A jaqueta de lona é presa a um suporte de alumínio, apoiado no braço de madeira que segura os pesos levantados pelo animal, e um estimulador elétrico foi conectado ao rabo do rato, de forma que ele recebesse um estímulo elétrico (10-15v, 0,3 s duração, 3 s intervalo).^12 , 20 - 22^

O período de treino durou 12 semanas e foi iniciado 48 horas após o teste 1RM. Cada sessão de treino de força foi realizada com 70% de sobrecarga de 1RM, com quatro sets de 12 repetições e intervalos de noventa segundos. Os animais do grupo de hipertensos somente receberam estímulo elétrico sem realizar o treino de força. O treinamento e a estimulação elétrica sempre foram realizados no início de ciclo ativo/escuro (18-20 horas), já que os animais apresentaram melhor tolerância ao exercício durante o ciclo escuro.^23^

### Aferição da pressão arterial (PA)

Vinte e quatro horas após o período de treinamento, os animais hipertensos foram testados novamente por 1RM, 48 horas após o teste de 1RM, a PA dos animais foi aferida. A PA dos animais foi medida pela implantação de um cateter na artéria femoral por meio de um transdutor de pressão (Edwards Lifescience, CA, EUA) acoplado a um pré-amplificador (BioData, Model BD-01, PB, Brasil).

Os sinais de PA pulsátil foram registrados por 30 minutos com os animais acordados (Advanced Codas/Windaq, Dataq Instruments Inc., OH, EUA), permitindo a análise de pulso-batimento-a-batimento para identificar a frequência cardíaca (FC), PA sistólica (PAS), e PA diastólica (PAD). A PA média (PAM) foi determinada pela PAS e pela DAP no próprio software de registro.

### Danos oxidativos

Após a avaliação da PA, os animais foram submetidos a eutanásia por decapitação sem anestesia,^24^ e o coração e o rim foram coletados para os testes de danos oxidativos e atividade de enzima antioxidante.

Para determinar os danos oxidativos a lipídios, os produtos de lipoperoxidação foram medidos por oxidação por xilenol laranja, em que a oxidação de íons ferrosos (Fe^2^) para íons férricos (Fe^3^) ocorre em condições ácidas, pelos lipídios de peróxidos de hidrogênio.^25^ Da mesma forma, o malondialdeído foi medido pela quantificação de substâncias reativas ao ácido tiobarbitúrico.^26^

Os grupos sulfidrílicos, que são estruturas associadas a proteínas e altamente suscetíveis a danos oxidativos, também foram medidos. Por meio de sua quantificação, é possível estimar o dano proteico nos tecidos. A determinação dos grupos sulfidrílicos foi feita pela reação entre o reagente de Ellman (DTNB) e sulfidrila livre da cadeia lateral de cisteína.^27^

### Atividade de enzima antioxidante

A atividade de SOD foi determinada pela habilidade da enzima do tecido de dissociar os ânions superóxidos derivados da auto-oxidação do pirogalol e sua reação reduzindo o 3-(4,5-dimetil-2-tiazolil )-2,5-difenil-2H-tetrazólio (MTT) e formando cristais de formazan.^26 , 28^

A atividade da CAT foi estimada pelo índice de degradação do peróxido de hidrogênio (H_2_O_2_) conforme o protocolo descrito previamente por Nelson e Kiesow.^29^ A atividade de GPx foi avaliada pela oxidação de NADPH, conforme descrito por Paglia e Valentine.^30^

### Determinação da concentração de proteína

A determinação de concentração de proteína nos testes realizados foi feita pela técnica de Lowry et al.,^31^ quantificando a concentração de proteínas presentes no homogenato das amostras, por comparação com uma curva padrão feita com albumina sérica.

### Análise estatística

A normalidade dos dados foi verificada utilizando-se o teste de normalidade Shapiro-Wilk. Os resultados são expressos em média ± desvio padrão (DP). A análise estatística foi realizada por meio de análise de variância (ANOVA) de um fator, seguida do teste de Bonferroni post-hoc. Um valor de p<0,05 foi considerado estatisticamente significativo. Foram realizadas análises estatísticas utilizando GraphPad Prism^TM^ 8.0.

## Resultados

Para validar nosso modelo de indução de hipertensão renovascular, foram avaliados parâmetros hemodinâmicos. Esses parâmetros foram medidos pela PA pulsátil com os animais acordados. A indução da hipertensão renovascular foi bem-sucedida e causou o aumento de PAS, PAD, PAM e FC. Por outro lado, o treino de força conseguiu neutralizar os efeitos da hipertensão renovascular ( Tabela 1 ).

**Tabela 1:** – Alteração na pressão arterial causada por estenose arterial renal

	Placebo	Hipertenso sedentário	Hipertenso treinado
PAS (mmHg)	133±2	187±5***	150±10^##^
PAD (mmHg)	92±1	151±6***	121±5**^,##^
PAM (mmHg)	114±2	165±5***	138±8*^,#^
FC (BPM)	337±4	385±9**	338±4^##^

Todos os dados representam média de ± EPM. *p<0,05, **p<0,01, ***p<0,001 em comparação com o placebo; #p<0,05, ##p<0,01 comparados com o grupo dos hipertensos sedentários, calculado por ANOVA de um fator seguido do teste de Bonferroni post hoc para comparações em pares. PAS: pressão arterial sistólica, PAD: pressão arterial diastólica, PAM: pressão arterial média, FC: frequência cardíaca, BPM: batimentos por minuto.

**Figura 2:**
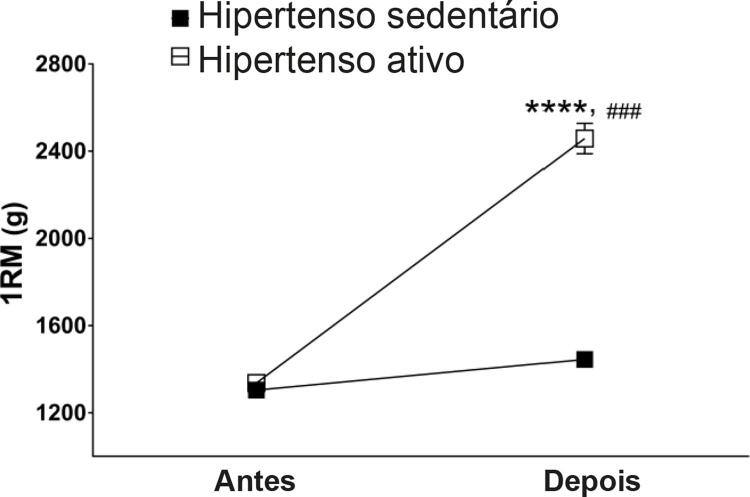
– Valores absolutos do teste de força máxima. Todos os dados representam média de ± EPM. ****p<0,0001 em comparação com antes do treino; ###p<0,001 comparado com o grupo dos hipertensos sedentários, calculado por ANOVA de dois fatores seguido do teste de Bonferroni post hoc para comparações em pares. 1RM: teste de repetição máxima.

Também avaliamos a eficiência do treino de força por uma medição de 1RM, que mede a força máxima dos animais. O treino de força promoveu o aumento na carga suspensa pelos animais hipertensos treinados após 12 semanas de treinamento (p<0,0001; Figura 2 ). Entretanto, como esperado, não houve mudanças na força dos ratos sedentários hipertensos (p>0,05).

**Figura 3:**
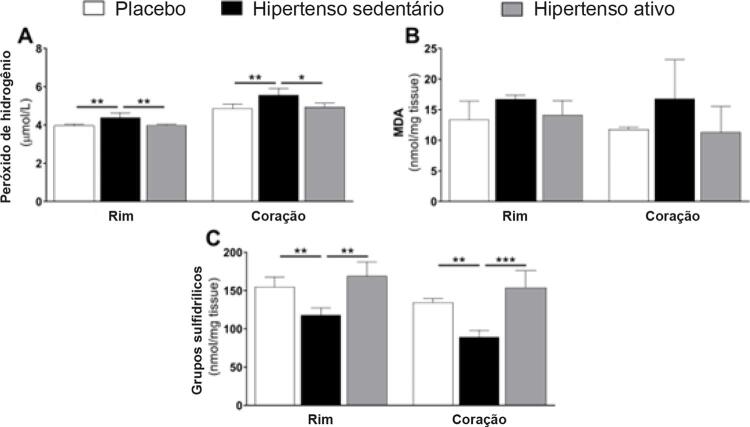
– Efeitos da hipertensão renovascular e do treino de força nos marcadores de danos oxidativos no rim contralateral e no coração. Todos os dados representam média de ± EPM. *p<0,05, **p<0,01, ***p<0,001, calculado por ANOVA de um fator seguido do teste de Bonferroni post hoc para comparações em pares. MDA: malondialdeído.

O aumento do stress oxidativo é outra marca da hipertensão. Dessa forma, medimos os danos oxidativos a lipídios e proteínas, medindo peróxidos de hidrogênio, malondialdeído, e grupos sulfidrílicos. Novamente foi possível validar nosso modelo de hipertensão, já que esta aumentou os danos a lipídios e proteínas no rim contralateral e no coração (p<0,01; Figura 3A e C ), pelo aumento dos peróxidos de hidrogênio e pela redução dos níveis de grupos sulfidrílicos. Entretanto, os animais treinados demonstraram ter proteção contra danos oxidativos com níveis baixos de peróxidos de hidrogênio e preservação dos grupos sulfidrílicos, no rim direito e no coração. Além disso, não se observaram mudanças no nível de malondialdeído (p>0,05; Figura 3B ).

**Figura 4:**
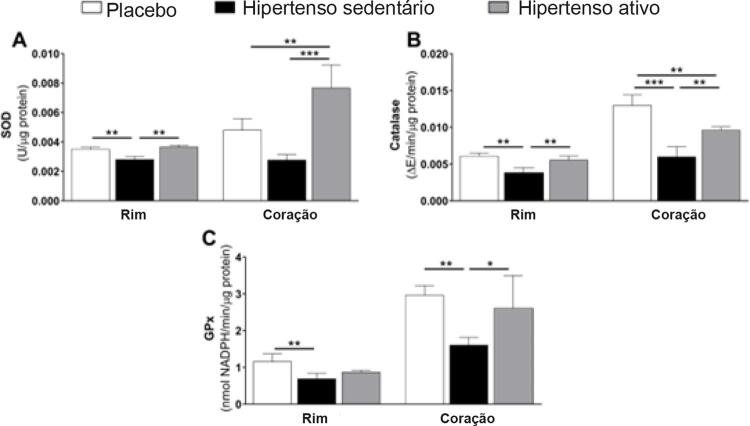
– Efeitos da hipertensão renovascular e do treino de força na atividade das enzimas antioxidantes. Todos os dados representam média de ± EPM. *p<0,05, **p<0,01, ***p<0,001, calculado por ANOVA de um fator seguido do teste de Bonferroni post hoc para comparações em pares. SOD: superóxido dismutase; Gpx: glutationa peroxidase.

Para melhor identificar os efeitos do treino de força no stress oxidativo em hipertensão renovascular, medimos a atividade das enzimas antioxidantes endógenas. O treino de força aumentou a atividade de SOD no coração e resgatou a atividade de SOD no rim (p<0,01; Figura 4A ), bem como a atividade da catalase em ambos os tecidos (p<0,01; Figura 4B ), enquanto a atividade da GPx só foi normalizada no coração (p<0,01; Figura 4C ).

## Discussão

Os principais resultados do presente estudo demonstraram que o treino de força de 12 semanas com intensidade moderada reduziu os danos oxidativos ao coração e rim contralateral na hipertensão renovascular, aumentando a atividade das enzimas antioxidantes endógenas, bem como reduzindo a pressão sanguínea.

Os modelos de hipertensão renovascular são conhecidos pela ativação do sistema renina-angiotensina, aumentando os níveis de angiotensina II, e com consequente aumento de PA.^16 , 17 , 32 , 33^ Como ocorreu no presente estudo, em que os animais passaram por indução de hipertensão tinham valores de PA elevados, demonstrando que o modelo de indução de hipertensão experimental foi realizado com sucesso.

Além disso, havíamos realizado o modelo de treino de força descrito por Tamaki, Uchiyama e Nakano,^19^ cujos efeitos benéficos semelhantes aos encontrados em seres humanos que praticam esse tipo de treino físico já foram relatados.^9 , 12 , 19 - 22 , 34^ No presente trabalho, identificou-se que um treino de força moderado foi eficiente no aumento da força dos animais treinados. Essa demonstração acarretou mudanças benéficas, como se pode ver pela redução da PA. Além disso, os efeitos benéficos também puderam ser observados pela redução de danos a lipídios e preservação dos grupos sulfidrílicos no coração e nos rins. Foi relatado na literatura que o treino de natação aeróbico realizado com intensidade moderada reduz os danos oxidativos no rim contralateral para estenose arterial renal.^35^

Outros estudos também demonstraram esse efeito protetor do exercício físico no stress oxidativo. Como foi relatado, o treinamento aeróbico em esteira com intensidade que aumenta progressivamente reduz os danos oxidativos renais em outro modelo de hipertensão experimental,^36^ bem como em outro modelo de doenças renais crônicas.^37^ Efeitos semelhantes também foram demonstrados em outros modelos de treino de força.^38 , 39^ Essa proteção promovida pelo exercício físico é importante para evitar a ocorrência de fibrose, um processo que ocorre pela deposição de colágeno nas áreas que sofreram danos oxidativos.^40^ Esses danos são aumentados na hipertensão renovascular devido à hiperativação do sistema renina-angiotensina-aldosterona, gerando stress oxidativo.^2 , 41^

Entretanto, o organismo tem mecanismos para evitar a ocorrência desses danos oxidativos, um dos quais sendo a ativação de enzimas antioxidantes endógenas.^42 , 43^ Por meio desse mecanismo, a enzima antioxidante SOD catalisa a dismutação de O_2_^-^ para H_2_O_2_. Em seguida, o H_2_O_2 _é reduzido para H_2_O e O_2 _pelas peroxidases, GPx ou CAT.^42 , 43^ Em indivíduos saudáveis, essas enzimas são expressas em diferentes maneiras em órgãos diferentes, dependendo dos processos metabólicos e funcionais que ocorrem neles. Entretanto, essas enzimas antioxidantes são reduzidas durante a hipertensão arterial.^44 , 45^

No presente estudo, a atividade reduzida das enzimas antioxidantes foi observada nos animais do grupo dos hipertensos. Outros estudos corroboram esses achados, demonstrando que tanto a atividade,^6^ quanto a expressão genética dessas enzimas são reduzidas nesse modelo de hipertensão renovascular.^5^ O treino de natação aeróbico^35 , 46^ demonstrou ter aumentado a atividade das enzimas SOD e CAT no coração e no rim contralateral de animais com hipertensão induzida, usando o mesmo modelo de hipertensão renovascular. Embora os efeitos do treino de força no stress oxidativo do rim contralateral ainda não tenham sido estudados, demonstrou-se que o treino de força em subida promove um aumento de enzimas antioxidantes nos músculos esquelético e cardíaco.^15 , 38 , 39^

Nosso estudo tem limitações porque, por motivos técnicos, não pudemos monitorar o decurso temporal da alteração na PA, bem como a medição de linha de base de outros parâmetros para entender melhor a ação terapêutica do treino de força. Apesar das limitações, os resultados demonstram, no modelo renovascular de ratos, que o treino de força tem um efeito protetor, como já foi observado em outras modalidades de exercício físico. O treino de força aumentou a atividade das enzimas SOD e CAT no rim contralateral e no coração, restabelecendo essa atividade antioxidante a valores encontrados em animais saudáveis (grupo placebo), indicando que esse é um mecanismo possível pelo qual o treino de força consegue reduzir danos oxidativos em animais com hipertensão renovascular.

## Conclusão

Os resultados encontrados no presente estudo nos permitem concluir que o treino de força pode neutralizar danos oxidativos produzidos por hipertensão renovascular no rim contralateral e no coração. Essa redução se deve, em parte, ao aumento da atividade das enzimas antioxidantes SOD e CAT promovido pelo treino de força. Portanto, esses resultados sugerem que o treino de força é uma ferramenta não farmacológica importante para o tratamento de hipertensão renovascular, com o potencial de evitar o avanço dos danos ao coração e rim que não tem estenose arterial renal.

## References

[1] . Kalra PA, Guo H, Kausz AT, Gilbertson DT, Liu J, Chen SC, et al. Atherosclerotic renovascular disease in United States patients aged 67 years or older: risk factors, revascularization, and prognosis. Kidney Int. 2005;68(1):293-301. 10.1111/j.1523-1755.2005.00406.x 15954920

[2] . Lerman LO, Textor SC, Grande JP. Mechanisms of tissue injury in renal artery stenosis: ischemia and beyond. Prog Cardiovasc Dis. 2009;52(3):196-203. 10.1016/j.pcad.2009.09.002 PMC2800096 19917330

[3] . Seshiah PN, Weber DS, Rocic P, Valppu L, Taniyama Y, Griendling KK. Angiotensin II stimulation of NAD(P)H oxidase activity: upstream mediators. Circ Res. 2002;91(5):406-13. 10.1161/01.res.0000033523.08033.16 12215489

[4] . Mervaala EM, Cheng ZJ, Tikkanen I, Lapatto R, Nurminen K, Vapaatalo H, et al. Endothelial dysfunction and xanthine oxidoreductase activity in rats with human renin and angiotensinogen genes. Hypertension. 2001;37(2 Pt 2):414-8. 10.1161/01.hyp.37.2.414 11230310

[5] . Nishi EE, Oliveira-Sales EB, Bergamaschi CT, Oliveira TG, Boim MA, Campos RR. Chronic antioxidant treatment improves arterial renovascular hypertension and oxidative stress markers in the kidney in Wistar rats. Am J Hypertens. 2010;23(5):473-80. 10.1038/ajh.2010.11 20186128

[6] . Toklu HZ, Sehirli O, Ersahin M, Suleymanoglu S, Yiginer O, Emekli-Alturfan E, et al. Resveratrol improves cardiovascular function and reduces oxidative organ damage in the renal, cardiovascular and cerebral tissues of two-kidney, one-clip hypertensive rats. J Pharm Pharmacol. 2010;62(12):1784-93. 10.1111/j.2042-7158.2010.01197.x 21054406

[7] . Vale AF, Carneiro JA, Jardim PCV, Jardim TV, Steele J, Fisher JP, et al. Acute effects of different resistance training loads on cardiac autonomic modulation in hypertensive postmenopausal women. J Transl Med. 2018;16(1):240. 10.1186/s12967-018-1615-3 PMC6117915 30165858

[8] . de Sousa EC, Abrahin O, Ferreira ALL, Rodrigues RP, Alves EAC, Vieira RP. Resistance training alone reduces systolic and diastolic blood pressure in prehypertensive and hypertensive individuals: meta-analysis. Hypertens Res. 2017;40(11):927-31. 10.1038/hr.2017.69 28769100

[9] . Pinter RCCE, Padilha AS, de Oliveira EM, Vassallo DV, Lizardo JHF. Cardiovascular adaptive responses in rats submitted to moderate resistance training. Eur J Appl Physiol. 2008;103(5):605-13. 10.1007/s00421-008-0761-3 18470531

[10] . Kuru O, Senturk UK, Kocer G, Ozdem S, Baskurt OK, Cetin A, et al. Effect of exercise training on resistance arteries in rats with chronic NOS inhibition. J Appl Physiol (1985). 2009;107(3):896-902. 10.1152/japplphysiol.91180.2008 19498093

[11] . Harris MB, Slack KN, Prestosa DT, Hryvniak DJ. Resistance training improves femoral artery endothelial dysfunction in aged rats. Eur J Appl Physiol. 2010;108(3):533-40. 10.1007/s00421-009-1250-z 19859729

[12] . Barauna VG, Batista Jr ML, Costa Rosa LF, Casarini DE, Krieger JE, Oliveira EM. Cardiovascular adaptations in rats submitted to a resistance-training model. Clin Exp Pharmacol Physiol. 2005;32(4):249-54. 10.1111/j.1440-1681.2005.04180.x 15810987

[13] . Araujo AJ, Santos AC, Souza KS, Aires MB, Santana-Filho VJ, Fioretto ET, et al. Resistance training controls arterial blood pressure in rats with L-NAME-induced hypertension. Arq Bras Cardiol. 2013;100(4):339-46. 23545992

[14] . Rodrigues MF, Stotzer US, Domingos MM, Deminice R, Shiguemoto GE, Tomaz LM, et al. Effects of ovariectomy and resistance training on oxidative stress markers in the rat liver. Clinics. 2013;68(9):1247-54. 10.6061/clinics/2013(09)12 PMC3782731 24141842

[15] . Scheffer DL, Silva LA, Tromm CB, da Rosa GL, Silveira PC, de Souza CT, et al. Impact of different resistance training protocols on muscular oxidative stress parameters. Appl Physiol Nutr Metab. 2012;37(6):1239-46. 10.1139/h2012-115 23176530

[16] . Goldblatt H, Lynch J, Hanzal RF, Summerville WW. Studies on Experimental Hypertension: I. The Production of Persistent Elevation of Systolic Blood Pressure by Means of Renal Ischemia. J Exp Med. 1934;59(3):347-79. 10.1084/jem.59.3.347 PMC2132360 19870251

[17] . Cangiano JL, Rodriguez-Sargent C, Martinez-Maldonado M. Effects of antihypertensive treatment on systolic blood pressure and renin in experimental hypertension in rats. J Pharmacol Exp Ther. 1979;208(2):310-3. 762665

[18] . American College of Sports Medicine (ACSM). ACSM’s guidelines for exercise testing and prescription. 9th ed. Philadelphia: Wolters Kluwer/Lippincott Williams & Wilkins; 2014.

[19] . Tamaki T, Uchiyama S, Nakano S. A weight-lifting exercise model for inducing hypertrophy in the hindlimb muscles of rats. Med Sci Sports Exerc. 1992;24(8):881-6. 1406173

[20] . Santana MNS, Souza DS, Miguel-Dos-Santos R, Rabelo TK, Vasconcelos CML, Navia-Pelaez JM, et al. Resistance exercise mediates remote ischemic preconditioning by limiting cardiac eNOS uncoupling. J Mol Cell Cardiol. 2018;125:61-72. 10.1016/j.yjmcc.2018.10.016 30339842

[21] . Macedo FN, Mesquita TR, Melo VU, Mota MM, Silva TL, Santana MN, et al. Increased Nitric Oxide Bioavailability and Decreased Sympathetic Modulation Are Involved in Vascular Adjustments Induced by Low-Intensity Resistance Training. Front Physiol. 2016;7:265. 10.3389/fphys.2016.00265 PMC4923192 27445854

[22] . Mota MM, Silva T, Macedo FN, Mesquita TRR, Quintans LJJ, Santana-Filho VJ, et al. Effects of a Single Bout of Resistance Exercise in Different Volumes on Endothelium Adaptations in Healthy Animals. Arq Bras Cardiol. 2017;108(5):436-42. 10.5935/abc.20170060 PMC5444890 28591321

[23] . Beck WR, Ribeiro LFP, Scariot PPM, dos Reis IGM, Gobatto CA. Time of day effects on aerobic capacity, muscle glycogen content and performance assessment in swimming rats. Science & Sports. 2014;29(6):319-23.

[24] . Leary S, Underwood W, Anthony R, Cartner S, Corey D, Greenacre C, et al. AVMA Guidelines for the Euthanasia of Animals. 13 ed. Schaumburg2013 2013.

[25] . Nourooz-Zadeh J, Tajaddini-Sarmadi J, Wolff SP. Measurement of plasma hydroperoxide concentrations by the ferrous oxidation-xylenol orange assay in conjunction with triphenylphosphine. Anal Biochem. 1994;220(2):403-9. 10.1006/abio.1994.1357 7978285

[26] . Britto RM, Silva-Neto JAD, Mesquita TRR, Vasconcelos CML, de Almeida GKM, Jesus ICG, et al. Myrtenol protects against myocardial ischemia-reperfusion injury through antioxidant and anti-apoptotic dependent mechanisms. Food Chem Toxicol. 2018;111:557-66. 10.1016/j.fct.2017.12.003 29208507

[27] . Faure P, Lafond JL. Measurement of plasma sulfhydryl and carbonyl groups as a possible indicator of protein oxidation. In: Favier AE, Cadet J, Kalyanaraman B, Fontecave M, Pierre JL, Editors. Analysis of Free Radicals in Biological Systems: Birkhäuser Basel; 1995. p. 237-48.

[28] . Madesh M, Balasubramanian KA. Microtiter plate assay for superoxide dismutase using MTT reduction by superoxide. Indian J Biochem Biophys. 1998;35(3):184-8. 9803669

[29] . Nelson DP, Kiesow LA. Enthalpy of decomposition of hydrogen peroxide by catalase at 25°C (with molar extinction coefficients of H_2_O_2_solutions in the UV). Anal Biochem. 1972;49(2):474-8. 10.1016/0003-2697(72)90451-4 5082943

[30] . Paglia DE, Valentine WN. Studies on the quantitative and qualitative characterization of erythrocyte glutathione peroxidase. J Lab Clin Med. 1967;70(1):158-69. 6066618

[31] . Lowry OH, Rosebrough NJ, Farr AL, Randall RJ. Protein measurement with the Folin phenol reagent. J Biol Chem. 1951;193(1):265-75. 14907713

[32] . Chrysoula B, Eleni G, Alexandros S, Alexandra K, Konstantinos C, Alexia P, et al. Renovascular Hypertension: Novel Insights. Curr Hypertens Rev. 2019;15:1-6.

[33] . Ceron CS, Rizzi E, Guimaraes DA, Martins-Oliveira A, Cau SB, Ramos J, et al. Time course involvement of matrix metalloproteinases in the vascular alterations of renovascular hypertension. Matrix Biology. 2012;31(4):261-70. 10.1016/j.matbio.2012.01.009 22342460

[34] . Ghiasi R, Mohammadi M, Ashrafi Helan J, Jafari Jozani SR, Mohammadi S, Ghiasi A, et al. Influence of Two Various Durations of Resistance Exercise on Oxidative Stress in the Male Rat’s Hearts. J Cardiovasc Thorac Res. 2015;7(4):149-53. 10.15171/jcvtr.2015.32 PMC4685280 26702343

[35] . Özdemir Kumral ZN, Şener G, Yeğen BÇ. Regular swimming exercise performed either before or after the induction of renovascular hypertension alleviates oxidative renal injury in rats. J Res Pharm. 2014;18(2):66-72.

[36] . Gu Q, Zhao L, Ma YP, Liu JD. Contribution of mitochondrial function to exercise-induced attenuation of renal dysfunction in spontaneously hypertensive rats. Mol Cell Biochem. 2015;406(1-2):217-25. 10.1007/s11010-015-2439-6 25963667

[37] . de Souza PS, da Rocha LG, Tromm CB, Scheffer DL, Victor EG, da Silveira PC, et al. Therapeutic action of physical exercise on markers of oxidative stress induced by chronic kidney disease. Life Sci. 2012;91(3-4):132-6. 10.1016/j.lfs.2012.06.028 22771699

[38] . Effting PS, Brescianini SMS, Sorato HR, Fernandes BB, Fidelis GdSP, Silva PRLd, et al. Resistance Exercise Modulates Oxidative Stress Parameters and TNF-α Content in the Heart of Mice with Diet-Induced Obesity. Arq Bras Cardiol. 2019;112:545-52. 10.5935/abc.20190072 PMC6555563 31038529

[39] . Neves RVP, Rosa TS, Souza MK, Oliveira AJC, Gomes GNS, Brixi B, et al. Dynamic, Not Isometric Resistance Training Improves Muscle Inflammation, Oxidative Stress and Hypertrophy in Rats. Front Physiol. 2019;10:4. 10.3389/fphys.2019.00004 PMC6349781 30723416

[40] . Zhong J, Guo D, Chen CB, Wang W, Schuster M, Loibner H, et al. Prevention of angiotensin II-mediated renal oxidative stress, inflammation, and fibrosis by angiotensin-converting enzyme 2. Hypertension. 2011;57(2):314-22. 10.1161/HYPERTENSIONAHA.110.164244 21189404

[41] . Nishi EE, Lopes NR, Gomes GN, Perry JC, Sato AYS, Naffah-Mazzacoratti MG, et al. Renal denervation reduces sympathetic overactivation, brain oxidative stress, and renal injury in rats with renovascular hypertension independent of its effects on reducing blood pressure. Hypertens Res. 2019;42(5):628-40. 10.1038/s41440-018-0171-9 30573809

[42] . Roumeliotis S, Roumeliotis A, Dounousi E, Eleftheriadis T, Liakopoulos V. Dietary Antioxidant Supplements and Uric Acid in Chronic Kidney Disease: A Review. Nutrients. 2019;11(8). 10.3390/nu11081911 PMC6723425 31443225

[43] . Ravarotto V, Simioni F, Pagnin E, Davis PA, Calò LA. Oxidative stress – chronic kidney disease – cardiovascular disease: A vicious circle. Life Sci. 2018;210:125-31. 10.1016/j.lfs.2018.08.067 30172705

[44] . Cardoso AM, Martins CC, Fiorin Fda S, Schmatz R, Abdalla FH, Gutierres J, et al. Physical training prevents oxidative stress in L-NAME-induced hypertension rats. Cell Biochem Funct. 2013;31(2):136-51. 10.1002/cbf.2868 22961602

[45] . Saravanakumar M, Raja B. Veratric acid, a phenolic acid attenuates blood pressure and oxidative stress in L-NAME induced hypertensive rats. Eur J Pharmacol. 2011;671(1-3):87-94. 10.1016/j.ejphar.2011.08.052 21937012

[46] . Maia RC, Sousa LE, Santos RA, Silva ME, Lima WG, Campagnole-Santos MJ, et al. Time-course effects of aerobic exercise training on cardiovascular and renal parameters in 2K1C renovascular hypertensive rats. Braz J Med Biol Res. 2015;48(11):1010-22. 10.1590/1414-431X20154499 PMC4671528 26270472

