# Supplementary material for: Treino de Força Reduz Stress Oxidativo Cardíaco e Renal em Ratos com Hipertensão Renovascular
Source: Arq Bras Cardiol. 2021 Jan 27;116(1):4–11. [Article in Portuguese] doi: 10.36660/abc.20190391 (PMC8159508; doi:10.36660/abc.20190391)
Supplement: Translation

# Translation: Strength Training Reduces Cardiac and Renal Oxidative Stress in Rats with Renovascular Hypertension

**Authors:** Rodrigo Miguel-dos-Santos, Jucilene Freitas dos Santos, Fabricio Nunes Macedo, Anderson Carlos Marçal, Valter J. Santana, Rogerio Brandão Wichi, Sandra Lauton-Santos

**Affiliations:** 1 Noruega Norwegian University of Science and Technology - Cardiac Exercise Reserch Group, Department of Circulation and Medical Imaging, Trondheim – Noruega; 2 Brazil Post-graduate Program of Physical Education, Federal University of Sergipe, São Cristóvão, SE – Brazil; 3 Brazil Post-graduate Program of Physiological Sciences, Federal University of Sergipe, São Cristóvão, SE – Brazil; 4 Brazil Institute of Biological Sciences and Health, Federal University of Alagoas, Maceió, AL – Brazil; 5 Brazil Department of Physical Education, Estacio University Center of Sergipe, Aracaju, SE – Brazil; 6 Brazil Department of Morphology, Federal University of Sergipe, São Cristóvão, SE – Brazil; 7 Brazil Post-graduate Program of Medicine, Federal University of Sergipe, São Cristóvão, SE – Brazil

**Keywords:** Hypertension, Renovascular, Resistance Training, Antioxidants, Oxidative Stress, Renal Arterial Obstruction, Oxidation-Reduction

## Abstract

**Background:**

Strength training has beneficial effects on kidney disease, in addition to helping improve antioxidant defenses in healthy animals.

**Objective:**

To verify if strength training reduces oxidative damage to the heart and contralateral kidney caused by the renovascular hypertension induction surgery, as well as to evaluate alterations in the activity of superoxide dismutase (SOD), catalase (CAT), and glutathione peroxidase (GPx) endogenous antioxidant enzymes.

**Methods:**

Eighteen male rats were divided into three groups (n=6/group): sham, hypertensive, and trained hypertensive. The animals were induced to renovascular hypertension through left renal artery ligation. Strength training was initiated four weeks after the induction of renovascular hypertension, continued for a 12-weeks period, and was performed at 70% of 1RM. After the training period, the animals were euthanized and the right kidney and heart were removed for quantitation of hydroperoxides, malondialdehyde and sulfhydryl groups, which are markers of oxidative damage. In addition, the activity of SOD, CAT, and GPx antioxidant enzymes was also measured. The adopted significance level was 5% (p < 0.05).

**Results:**

After strength training, a reduction in oxidative damage to lipids and proteins was observed, as could be seen by reducing hydroperoxides and total sulfhydryl levels, respectively. Furthermore, an increased activity of superoxide dismutase, catalase, and glutathione peroxidase antioxidant enzymes was observed.

**Conclusion:**

Strength training is able to potentially reduce oxidative damage by increasing the activity of antioxidant enzymes. (Arq Bras Cardiol. 2021; 116(1):4-11)

## Introduction

Renovascular hypertension, a type of hypertension caused by total or partial renal artery stenosis due to genetic factors or atherosclerosis, is an important cause of secondary hypertension.^1^In this type of hypertension, the increase in arterial pressure (AP) is triggered by the greater release of renin by the ischemic kidney as a result of the reduction of blood flow to this organ, due to the stenosis of the renal artery.^1 , 2^

Renin is responsible for the conversion of angiotensinogen to angiotensin I, which is cleaved by the angiotensin-converting enzyme (ACE), producing angiotensin II (Ang II).^3 , 4^Thus, the elevation of renin triggers an increase in Ang II release. Ang II, in turn, activates the NADPH oxidase^3^and xanthine oxidase^4^enzymes, increasing the production of superoxide anion (O_2_^-^), a highly reactive pro-oxidant signaling molecule that can cause oxidative damage to lipids, proteins, and DNA, as has been described in renovascular hypertension.^5 , 6^Increased oxidative damage in the kidney and heart may lead to increased fibrosis of the tissue, leading to a reduction of its function,^2^and, eventually, leading to the failure of the kidney that was not affected by stenosis and cardiac dysfunction.

It is reported in the literature the protective action of strength training in the treatment of several diseases, among them arterial hypertension.^7 , 8^Among the benefits generated by strength training, it has already been seen that it promotes the improvement of the cardiac function,^9^as well as increased activity and/or expression of the enzymes involved with the synthesis of nitric oxide.^10 , 11^These changes result in an increased release of nitric oxide, an improvement of vascular tone,^10 , 11^and a reduction in AP in normotensive^12^and hypertensive animals.^13^

In addition, reports in the literature have also described the protective action of strength training in oxidative stress, improving the antioxidant defense in the liver^14^and skeletal muscle.^15^However, the effects of strength training on the heart and contralateral kidney to renal artery stenosis are unknown. Hence, the present study sought to verify if strength training reduces the oxidative damage to the heart and contralateral kidney caused by renovascular hypertension induction surgery, as well as to evaluate the alterations in the activity of the superoxide dismutase (SOD), catalase (CAT), and glutathione peroxidase (GPx) endogenous antioxidant enzymes.

## Methods

The experimental protocol of the present study was approved by the Animal Research Ethics Committee (CEPA - #54/2015) of the Federal University of Sergipe, in compliance with the Ethical Principles of Animal Experimentation adopted by the National Council for Animal Experimentation Control (CONCEA).

### Sample

Male Wistar rats aged 10 to 12 weeks and body mass between 240 and 270 g were obtained from the animal facility of the Federal University of Sergipe. The animals were housed in collective cages (five animals/cage), kept under controlled temperature conditions (23 ± 1ºC) and a light-dark cycle of 12 hours, with feed and water ad libitum.

### Experimental groups

Eighteen animals were randomly divided, through an online software, into three experimental groups (n = 6 per group): sham, hypertensive, and trained hypertensive. The sample size was defined by convenience.

### Renovascular hypertension induction

Induction to hypertension was performed in the animals from the hypertensive and trained hypertensive groups, applying the renal artery clipping model, developed by Goldblatt et al.,^16^following the adaptations proposed by Cangiano et al.^17^Thus, with animals under deep anesthesia (ketamine 90 mg/kg and xylazine 10 mg/kg, intraperitoneal), an incision was made in the left flank of the animals’ back to exteriorize the left kidney, and a ligation of the renal artery was performed with a 4.0 sterile cotton surgical line. The animals of the Sham group underwent surgery only to exteriorize the left kidney so as to mimic the stress generated by the surgery in the animals from the hypertensive and trained hypertensive groups. All animals received painkillers (Flunixin meglumine, sc, 1 mg/Kg, every 24h) for four days following post-surgery.

### Strength training protocol

Three weeks after the hypertension induction surgery, the animals from the hypertensive and trained hypertensive groups were adapted to the training apparatus for five days, keeping the animals attached to the equipment for 10 minutes each day. Thereafter, a maximum repetition test (1RM) was performed in the animals of both groups and every two weeks in the trained hypertensive group, in order to determine the load used in the training sessions. The test was performed again in the sedentary hypertensive group at the end of the experimental protocol only.

The maximum repetition tests were performed following the American College of Sports Medicine guidelines^18^for humans, with three attempts per test. The first 1RM test was performed with 3x the animal body weight, adjusting up or down for the next try depending on the animal’s performance in the attempt. The animals were allowed to rest for three minutes between each try.

Strength training was performed as described by Tamaki, Uchiyama, and Nakano,^19^and as used in other studies.^20 - 22^Briefly, this strength training model is performed in a squat-mimetic apparatus, where the torso of rats is fitted with a canvas jacket keeping them in an upright position ( Figure 1 ). The canvas jacket was attached to an aluminum bracket, which is held by the wooden arm holding weights for the animals to lift, and an electro-stimulator was connected to their tail in such a way that the animals received an electrical stimulus (10-15v, 0.3s duration, 3s interval).^12 , 20 - 22^

**Figure 1:** – Representative illustration of strength training apparatus. (Adapted from Tamaki et al., 1992).

The training period lasted 12 weeks and was started 48 hours after the 1RM test. Each strength training session was done with a 70% overload of 1RM, with four sets of 12 repetitions, and ninety-second intervals. The animals of the hypertensive group received only electrical stimulation without performing strength training. Training and electrostimulation were always performed at the beginning of the active/dark cycle (18-20 h), as it is during the dark cycle that the animals presented better tolerance to exercise.^23^

### Arterial pressure (AP) measurement

Twenty-four hours after the training period, the hypertensive animals were again tested for 1RM and, 48 hours after the test of 1RM, the AP of the animals was measured. The AP of the animals was measured by implantation of a catheter in the femoral artery through a pressure transducer (Edwards Lifescience, CA, USA) attached to a preamplifier (BioData, Model BD-01, PB, Brazil).

The pulsatile AP signals were recorded for 30 minutes with the animals awake (Advanced Codas/Windaq, Dataq Instruments Inc., OH, USA), allowing pulse-beat-to-beat analysis to identify heart rate (HR), systolic AP (SAP), and diastolic AP (DAP). The mean AP (MAP) was determined through SAP and DAP in the recording software itself.

### Oxidative damage

After the AP evaluation, the animals were euthanized by decapitation without anesthesia,^24^and the heart and right kidney were harvested for the oxidative damage and antioxidant enzyme activity assays.

To determine oxidative damage to lipids, the products of lipoperoxidation were measured by oxidation of xylenol orange, in which the oxidation of ferrous ions (Fe^2^) to ferric ions (Fe^3^) occurs under acidic conditions, by the hydroperoxides lipids.^25^In addition, malondialdehyde was measured by the quantification of the thiobarbituric acid reactive substances.^26^

Sulfhydryl groups, which are structures associated with proteins and are highly susceptible to oxidative damage, have also been measured. Through its quantification, it is possible to estimate the protein damage in the tissues. The determination of sulfhydryl groups was performed by reacting 5’5-dithio-bis-2-nitrobenzoic acid (DTNB) with free sulfhydryl of the cysteine side chain.^27^

### Antioxidant enzyme activity

SOD activity was determined by the ability of the tissue enzyme to dissociate the superoxide anions derived from pyrogallol self-oxidation and their reaction reducing 3-(4,5-dimethylthiazol-2-yl)-2,5-diphenyltetrazolium (MTT) and forming formazan crystals.^26 , 28^

CAT activity was estimated by the rate of degradation of hydrogen peroxide (H_2_O_2_) according to the protocol previously described by Nelson and Kiesow.^29^GPx activity was assessed by oxidation of NADPH, as described by Paglia and Valentine.^30^

### Determination of protein concentration

The protein concentration was determined in this study’s tests by applying the technique set forth by Lowry et al.,^31^quantifying the concentration of proteins present in the homogenate of the samples by comparing this to a standard curve made with serum albumin.

### Statistical analysis

The normality of the data was verified by applying the Shapiro-Wilk normality test. Results are expressed as mean ± standard deviation (SD). Statistical analysis was performed through the one-way analysis of variance (ANOVA), followed by the Bonferroni post-hoc test. A value of p<0.05 was considered as statistically significant. Statistical analyses were performed using the GraphPad Prism^TM^8.0.

## Results

To validate our model of renovascular hypertension induction, hemodynamic parameters were assessed. These parameters were measured through the pulsatile AP with the animals awake. The induction of renovascular hypertension was successful and caused the increase of SAP, DAP, MAP, and HR, whereas the strength training was able to counteract the effects of renovascular hypertension ( Table 1 ).

**Table 1:** – Arterial pressure alteration caused by renal artery stenosis

	Sham	Hypertensive sedentary	Hypertensive trained
SAP (mmHg)	133±2	187±5***	150±10^##^
DAP (mmHg)	92±1	151±6***	121±5**^,##^
MAP (mmHg)	114±2	165±5***	138±8*^,#^
HR (BPM)	337±4	385±9**	338±4^##^

All data represent mean ± SEM. *p<0.05, **p<0.01, ***p<0.001 compared with sham; #p<0.05, ##p<0.01 compared with hypertensive sedentary, calculated by one-way ANOVA followed by the post hoc Bonferroni test for pairwise comparisons. SAP: systolic arterial pressure, DAP: diastolic arterial pressure, MAP: mean arterial pressure, HR: heart rate, BPM: beats per minute.

We also evaluated the effectiveness of strength training through the measurement of 1RM, which measures the maximum strength of the animals. Strength training promoted an increase in the load lifted by the trained hypertensive animals after 12 weeks of training (p<0.0001; Figure 2 ). Nonetheless, as expected, there was no change in the strength of the sedentary hypertensive rats (p>0.05).

**Figure 2:** – Absolute values of the maximum strength test. All data represent mean ± SEM. ****p<0.0001 compared with before training; ###p<0.001 compared with hypertensive sedentary before, calculated by two-way ANOVA followed by the post hoc Bonferroni test for pairwise comparisons. 1RM: maximum repetition test.

Increased oxidative stress is another hallmark of hypertension. In this light, we measured the oxidative damage to lipids and proteins by measuring hydroperoxides, malondialdehyde, and sulfhydryl groups. Again, it was possible to validate our model of hypertension since hypertension increased the damage to lipids and proteins in the contralateral kidney and heart (p<0.01; Figure 3A and C ), through the increase of hydroperoxides and reduction of sulfhydryl group levels. However, trained animals showed protection against oxidative damage with low levels of hydroperoxides and the preservation of sulfhydryl groups in both the right kidney and the heart. In addition, no significant change was observed in the level of malondialdehyde (p>0.05; Figure 3B ).

**Figure 3:** – Effects of renovascular hypertension and strength training on the markers of oxidative damage in the contralateral kidney and heart. All data represent mean ± SEM. *p<0.05, **p<0.01, ***p<0.001, calculated by one-way ANOVA followed by the post hoc Bonferroni test for pairwise comparisons. MDA: malondialdehyde.

To further identify the effects of strength training on oxidative stress in renovascular hypertension, the activity of the endogenous antioxidant enzymes was measured. Strength training increased SOD activity in the heart and rescued SOD activity in the kidney (p<0,01; Figure 4A ), as well as catalase activity in both tissues (p<0,01; Figure 4B), whereas GPx activity was only normalized in the heart (p<0,01; Figure 4C ).

**Figure 4:** – Effects of renovascular hypertension and strength training on the antioxidant enzyme activity. All data represent mean ± SEM. *p<0.05, **p<0.01, ***p<0.001, calculated by one-way ANOVA followed by the post hoc Bonferroni test for pairwise comparisons. SOD: superoxide dismutase; GPx: glutathione peroxidase.

## Discussion

The main results of the present study demonstrated that 12-week strength training with a moderate intensity reduced oxidative damage to the heart and contralateral kidney in renovascular hypertension by increasing the activity of endogenous antioxidant enzymes as well as by reducing blood pressure.

Renovascular hypertension models are well-known for renin-angiotensin system activation, increasing angiotensin II levels and consequent increases in AP.^16 , 17 , 32 , 33^As occurred in the present study, the animals that underwent hypertension induction presented elevated AP values, demonstrating that the experimental hypertension induction model was successfully performed.

Furthermore, the strength training model was performed, as described by Tamaki, Uchiyama and Nakano,^19^which has been reported to show beneficial effects that are similar to those found in humans who practice this type of physical training.^9 , 12 , 19 - 22 , 34^In the present work, it was found that moderate strength training was efficient in increasing the strength of the trained animals. Demonstrating that triggered beneficial changes, as was also seen by the reduction of AP. In addition, the beneficial effects could also be observed by reducing lipid damage and preserving the sulfhydryl groups in the heart and kidney. It has been reported in the literature that aerobic swimming training performed with moderate intensity reduces oxidative damage in the kidney contralateral to renal artery stenosis.^35^

Other studies have also demonstrated this protective effect of physical exercise on oxidative stress. As has been reported, aerobic treadmill training with progressively increasing intensity reduces renal oxidative damage in other models of experimental hypertension,^36^as well as in another models of chronic kidney diseases.^37^Similar effects have been also shown in other strength training models.^38 , 39^This protection promoted by physical exercise is important to prevent the occurrence of fibrosis, a process that occurs through the deposition of collagen in the areas that suffered oxidative damage.^40^These damages are increased in renovascular hypertension due to the hyperactivation of the renin angiotensin aldosterone system, generating oxidative stress.^2 , 41^

However, the organism has mechanisms to prevent the occurrence of these oxidative damages; one of these mechanisms occurs through the activation of the endogenous antioxidant enzymes.^42 , 43^By means of this mechanism, the antioxidant enzyme SOD catalyzes the dismutation of O_2_^-^to H_2_O_2_. Subsequently, the H_2_O_2_is reduced to H_2_O and O_2_by the peroxidases, GPx, or CAT.^42 , 43^In healthy individuals, these enzymes are expressed in different ways in different organs, depending on the metabolic and functional processes that occur in them. Nevertheless, these antioxidant enzymes are reduced during arterial hypertension.^44 , 45^

In the present study, reduced activity of antioxidant enzymes was observed in the animals from the hypertensive group. Other studies corroborate these findings, showing that both the activity^6^and the gene expression of these enzymes are reduced in this model of renovascular hypertension.^5^Aerobic swimming training^35 , 46^has been shown to increase the activity of SOD and CAT enzymes in the heart and contralateral kidney of animals with induced hypertension, using the same renovascular hypertension model. Although the effects of strength training on contralateral kidney oxidative stress have not yet been studied, it has been shown that climbing strength training promotes an increase in antioxidant enzymes in skeletal and cardiac muscles.^15 , 38 , 39^

This study presents limitations since, for technical reasons, we were not able to monitor the time-course of change in AP not the baseline measurement of other parameters for a better understanding of the therapeutical action of strength training. Despite the limitations, our results demonstrate, in a rat renovascular model, that strength training has a protective effect, as has already been observed in other modalities of physical exercise. Strength training increased the activity of SOD and CAT enzymes in the contralateral kidney and heart, reestablishing this antioxidant activity to values found in healthy animals (Sham group), indicating that this is a possible mechanism by which strength training is able to reduce oxidative damage in renovascular hypertensive animals.

## Conclusion

The results found in the present study allow us to conclude that strength training is able to counteract oxidative damage produced by renovascular hypertension in the contralateral kidney and heart. This reduction is due, in part, to the increased activity of the antioxidant enzymes SOD and CAT promoted by strength training. Therefore, these results suggest that strength training is an important non-pharmacological tool for the treatment of renovascular hypertension, potentially preventing the progression of damage to the heart and kidney without renal artery stenosis.
